# A Comparative Analysis of Machine Learning with WorldView-2 Pan-Sharpened Imagery for Tea Crop Mapping

**DOI:** 10.3390/s16050594

**Published:** 2016-04-26

**Authors:** Yung-Chung Matt Chuang, Yi-Shiang Shiu

**Affiliations:** Department of Urban Planning and Spatial Information, Feng Chia University, Taichung 40724, Taiwan; yungcchuang@fcu.edu.tw

**Keywords:** WorldView-2, tea crops, GLCM texture, pixel and object-based image analysis, random forest, support vector machine

## Abstract

Tea is an important but vulnerable economic crop in East Asia, highly impacted by climate change. This study attempts to interpret tea land use/land cover (LULC) using very high resolution WorldView-2 imagery of central Taiwan with both pixel and object-based approaches. A total of 80 variables derived from each WorldView-2 band with pan-sharpening, standardization, principal components and gray level co-occurrence matrix (GLCM) texture indices transformation, were set as the input variables. For pixel-based image analysis (PBIA), 34 variables were selected, including seven principal components, 21 GLCM texture indices and six original WorldView-2 bands. Results showed that support vector machine (SVM) had the highest tea crop classification accuracy (OA = 84.70% and KIA = 0.690), followed by random forest (RF), maximum likelihood algorithm (ML), and logistic regression analysis (LR). However, the ML classifier achieved the highest classification accuracy (OA = 96.04% and KIA = 0.887) in object-based image analysis (OBIA) using only six variables. The contribution of this study is to create a new framework for accurately identifying tea crops in a subtropical region with real-time high-resolution WorldView-2 imagery without field survey, which could further aid agriculture land management and a sustainable agricultural product supply.

## 1. Introduction

In recent years, due to global warming, many regions of the Earth have suffered from drastic climate change, which has increased the frequency of extreme climate events. Not only does this cause certain inconveniences for the lives of human beings, but it also threatens our lives and property. The growth of crops has an intimate relationship with the climate, so any climatic change could impact the amount and the quality of crop production [1,2,3,4,5,6,7,8,9,10,11]. In the past, in order to control the production surface area and the amount of production, governments would generally monitor the sensitive crops with prices that fluctuate according to the weather, disasters, or public preferences so as to prevent price collapses, which could cause financial damage to farmers. Therefore, it is important to have a comprehensive understanding of the acreage of crop production and the market situation of various crops in order to propose important contingency measures in response to the economic losses and potential food crises caused by extreme climate phenomena. In the sub-tropical region in Taiwan, for example, tea is a sensitive crop of high economic value and a featured export product. About 3306 tons of teas of various special varieties are exported annually, and the number is still growing. According to the United Nations Food and Agriculture Organization (FAO), the global production of tea grew from 3.059 million tons in 2001 to 4.162 million tons in 2010, while the global area planted with tea grew from 2.41 million hectares in 2001 to 3.045 million hectares in 2010. This is certainly an amazing growth rate, and many studies conducted in different countries found that global warming would result in tea production losses [12,13,14,15], so adopting effective measures for productivity control is necessary.

Timely and accurate LULC interpretation and dynamic agriculture structure monitoring are the key points of agricultural productivity control to ensure a sustainable food supply [16,17,18,19,20,21,22]. In actual operation, on-site investigation and orthogonal photograph interpretation are two methods widely used, especially by government agencies, to obtain information about tree characteristics and crop species composition. However, on-site investigation requires a lot of time and manpower and even though the working process is very detailed and precise, the ineffective investigation results in incomplete, unstable and sporadic LULC products. Aerial photograph interpretation and vector digitizing on GIS platforms (e.g., ArcGIS, MapInfo, QGIS) is also time-consuming and expensive for large crop mapping and classification. Besides, it is easier for the researchers to obtain extensive LULC information with high temporal resolution by using satellite imagery rather than aerial photographs or other methods. This information is fundamental for agricultural monitoring because the farmer compensation and the recognition of production losses are highly time-dependent. Therefore, daily transmission satellite imagery could be a very useful tool for LULC mapping.

However, the accurate interpretation ability for individual stands with the highly heterogeneous farm or shape characters of single canopies, is limited by the relatively low spatial resolution if we apply moderate-resolution satellite imagery (e.g., SPOT 4 and SPOT 5 provided by Airbus Defence and Space) [23,24,25,26,27,28,29,30,31]. Over the last few years, the spectral resolution, spatial resolution and temporal resolution have undergone rapid and substantial progress (e.g., Formosat-2 multispectral images offer 8 m resolution and panchromatic band images at 2 m resolution). For some urban and flat areas, individual stands, such as a tree or a shrub, can be easily distinguished by using very high resolution (VHR) imagery [32,33,34,35]. This provides opportunities to differentiate farm and crop types. High spatial resolution satellite imagery has thus become a cost-effective alternative to aerial photography for distinguishing individual crops or tree shapes [36,37,38,39,40,41,42,43,44,45,46]. For example, WorldView-2 images and support vector machine classifier (SVM) have been used to identified three dominant tree species and canopy gap types (accuracy = 89.32.1%) [47]. Previous study has applied a reproducible geographic object-based image analysis (GEOBIA) methodology to find out the location and shape of tree crowns in urban areas using high resolution imagery, and the identification rates were up to 70% to 82% [36]. QuickBird image classification with GEOBIA has also been proved useful to identify urban LULC classes and obtained a high overall accuracy (90.40%) [32].

Apart of the impact of satellite imagery resolution, image segmentation methods and classification algorithms also play essential roles in LULC classification workflows. In early years, many studies focused on developing pixel-based image analysis (PBIA) by taking the advantage of VHR image characteristics [48,49,50,51,52,53,54], but it is obvious that recent studies mapping vegetation composition or individual feature have demonstrated the advantages of object-based image analysis (OBIA) [55,56,57,58,59,60,61,62]. In OBIA, researchers first have to transfer the original pixel-image into object units based on specific arrangement or image characteristics, which is called image segmentation, and then assign the category of every object unit by using classifiers. OBIA can improve the deficiency of PBIA, such as the salt and pepper effect, therefore it has become one of the most popular workflows. For example, Ke and Quackenbush classified five tree species (spruce, pine, hemlock, larch and deciduous) in a sampled area of New York (USA) by applying object-based image analysis with QuickBird multispectral imagery and obtained an average accuracy of 66% [63]. Pu and Landry presented a significantly higher classification accuracy of five vegetation types (including broad-leaved, needle-leaved and palm trees) using object-based IKONOS image analysis [39]. The results were much better than using pixel-based approaches. Kim *et al.* obtained a higher overall accuracy of 13% with object-based image analysis by using an optimal segmentation of IKONOS image data to identify seven tree species/stands [64]. Ghosh and Joshi compared several classification algorithms on mapping bamboo patches using very high resolutionWorldView-2 imagery, and achieved 94% producer accuracy with an object-based SVM classifier [65].

According to the abovementioned reasons and background, the main objectives of this study include: (1) to investigate the classification accuracy of a combined workflow applied on WorldView-2 imagery to identify tea crop LULC; (2) to compare the capabilities of several ordinary LULC classification algorithms (e.g., maximum likelihood (ML), logistic regression (LR) random forest (RF) and support vector machine (SVM) based on pixel and object-based domains) in identifying tea LULC; and (3) to explore the potential of using high spatial/spectral/time resolution WorldView-2 imagery to identify tea crop LULC classification, and the details about image segmentation and feature extraction will also be discussed. The main content of this paper is divided into four major sections. Section 1 and Section 2 include descriptions and introductions of the study, introductions to on site investigation and satellite imagery data sets, frameworks of feature extraction and classification developed for this study. Section 3 contains the main findings and breakthroughs of this study, followed by their discussion. The final part consists of the conclusions.

## 2. Materials and Methods

### 2.1. Study Area and Workflow

**Figure 1:**
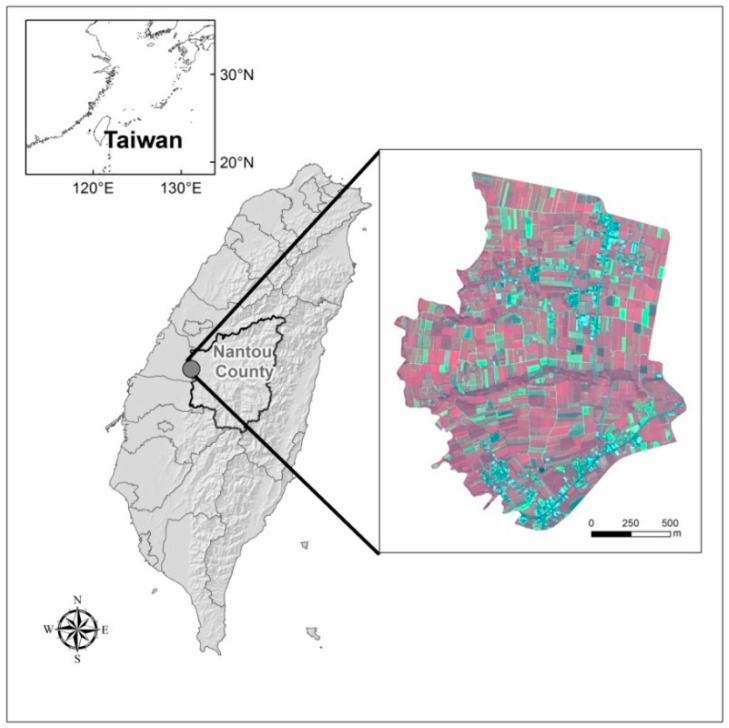
The location of study area in Nantou, Taiwan and the WorldView-2 imagery used to map tea cultivation area in this study.

**Figure 2:**
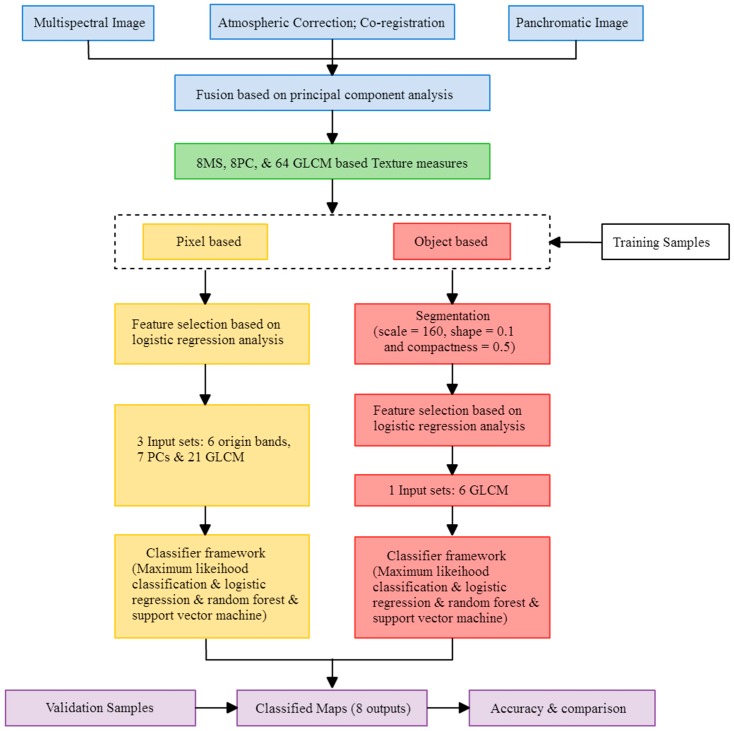
Methodological flowchart for mapping tea cultivation area.

The study area is located in part of Nantou County in central Taiwan (between 120.620°E to 120.683°E and 23.8492°N to 23.8894°N). The total area is about 263 hectares. According to the 2014 agricultural statistic data of Taiwan, the annual tea production of this area is about 2.3% of the total output. The climate of this region is sub-tropical, with average temperatures of 22~25 °C and an annual rainfall of 1500~2100 mm. The terrain is mainly a flat plain near low slope hills. This region contains typical agricultural villages with abundant and minimally changed *Camellia sinensis* (L.) *O. Ktze. Var. Sinensis* and *Camellia sinensis* (L.) *O. Ktze. Var. Assamica (Mast.) Kitam. f. Assamica* tea crops and pineapple LULC. The study area is shown in Figure 1. The methodological flowchart for mapping the tea cultivation area is shown in Figure 2.

### 2.2. Data Sets

#### 2.2.1. Collection of Ground Reference Data

**Figure 3:**
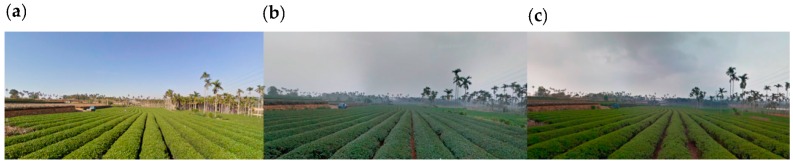
Tea crops (*Camellia sinensis* (L.) *O. Ktze. Var. Sinensis* and *Camellia sinensis* (L.) *O. Ktze. Var. Assamica* (*Mast.*) *Kitam. f. Assamica*) of three different seasons located in study area. (**a**) December 2014; (**b**) March 2015; (**c**) August 2015.

We surveyed the exact plantation location of all tea and other non-tea LULC during 4–5 March 2015 (Figure 3). A Garmin global positioning system (GPS) and ArcGIS mobile device were used as the tools for semi-automatic site mapping with a scale of 1:2500. The LULC includes five types: (1) tea (*Camellia sinensis* (L.) *O. Ktze. Var. Sinensis* and *Camellia sinensis* (L.) *O. Ktze. Var. Assamica* (*Mast.*) *Kitam. f. Assamica*); (2) other crops (e.g., pineapple and betel palm (*Areca catechu*)); (3) water and ponds; (4) buildings and roads; and (5) forest (evergreen broadleaf forest). Other crops in the study area were mainly pineapple and betel nut. We re-categorized these five LULC types into “tea” and “non-tea” LULC by integrating all land features except the tea areas. The results of the field survey showed that there are around 33 hectares of tea and 230 hectares of non-tea regions in our study area.

#### 2.2.2. WorldView-2 Imagery

**Figure 4:**
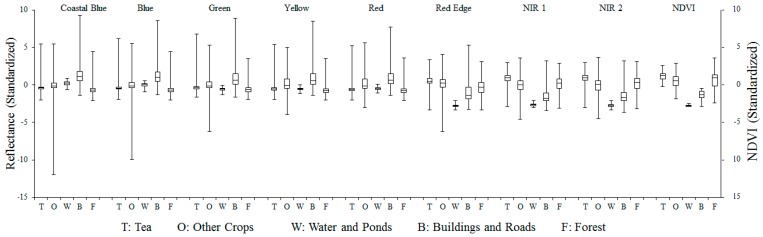
Distribution of standardized reflectance of different LULC across 8 multi-spectral bands and NDVI.

The main LULC of the study area are tea crops and pineapples, but it was not easy to do artificial interpretation even with VHR orthogonal aerial photographs due to the similar arrangements of the plants. For this reason, WorldView-2 imagery with both high spatial and spectral resolution was applied as a main material. The WorldView-2 imagery satellite (DigitalGlobe, Inc., Westminster, CO, USA) is a commercial satellite with eight multispectral bands and very high resolution. The average amount of time needed to revisit and acquire data for the exact same location is 1.1 day, and the spatial resolution is 0.46~0.52 m (panchromatic band, 450~800 nm) and 1.84~2.08 m (multispectral bands). The eight multispectral bands contained coastal blue (400~450 nm), blue (450~510 nm), green (510~580 nm), yellow (585~625 nm), red (630~690 nm), red edge (705~745 nm), NIR1 (770~895 nm) and NIR2 (860~1040 nm). The added spectral diversity of WorldView-2 satellite such as coastal blue, yellow and red edges provides the ability to perform detailed environmental and landscape change detection better than with other satellite images (e.g., Formosat-2, SPOT6, Kompsat) and DMC aerial photographs with only four bands or less. We applied cloud-free imagery (<5%) of the study area as the original material. The original digital number (DN) value of the imagery was converted to top-of-atmosphere spectral reflectance [66]. The imagery was also ortho-rectified and co-registered to the two degree zone Transverse Mercator projection coordinate system with a root mean square error of less than 0.5 pixels per image. The time of image acquisition for this study was acquired on 22 February 2015 corresponding to the date of ground reference data (4–5 March 2015). It worth mentioned that tea crops within study area are stable in LULC, farm shape arrangement, and not leafing out all years (Figure 4). Therefore, if the tea LULC still remained, the image acquisition and interpretation would not be limited or affected by season or date within a period due to stable vegetation spectral dynamics. This is beneficial to ground verification and long term monitoring.

As for the resolution of WorldView-2 imagery, the intervals between every tea plantation were not clear in the 2 m resolution MS bands in this study. Therefore, a pan-sharpening process was added to improve the spatial resolution of the MS bands from 2 m to 0.5 m. The pan-sharpened archives had enhanced interpretability in inferring forms and shapes of tea crop LULC. Each band of the pan-sharpened imagery was then standardized by using mean and standard deviation. The reason is that the reflectance value range of the WorldView-2 image varies between each band and makes the input variables contribute unequally to the PBIA and OBIA.

### 2.3. Spectral Variability Analysis

The common scientific challenge of mapping vegetation with very high spatial resolution imagery is to distinguish between different vegetation classes due to the superimposition of spectral signatures. In order to test the LULC classification capability of eight MS band WorldView-2 imagery, and the difference or similarity of tea crops in different season, spectral separability analysis was undertaken as the initial step. Figure 4 presents the box-whisker-plots of spectral variability of the training pixels of five basic LULC classes. Reflectance of buildings and roads in NIR1, NIR2, coastal blue wavelengths and reflectance of water and ponds in all red edge wavelengths were significantly different from other classes. Besides, in most bands, tea LULC, forest canopies, and other crops (pineapple crop and betel palm) showed similar spectral variability in the blue, green, yellow, red bands and normalized difference vegetation index (NDVI, ranges from minus one (−1) to plus one (+1)), and little difference in NIR1 and NIR2 bands. In other words, tea farm patches cannot be definitely distinguished from non-tea crops if we only use eight MS bands and NDVI without other information.

**Figure 5:**
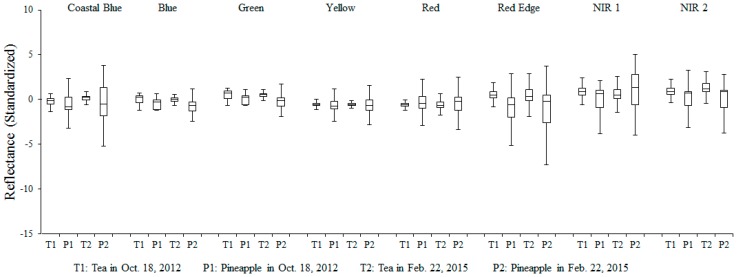
Standardized reflectance of tea crops (*Camellia sinensis* (L.) *O. Ktze. Var. Sinensis* and *Camellia sinensis* (L.) *O. Ktze. Var. Assamica* (*Mast.*) *Kitam. f. Assamica*) and pineapple in different dates across eight multi-spectral bands.

As for the evaluation of tea spectral dynamics, usually, when plants are harvested, they have a physiological response characterized by intensive vegetation growth and high primary production to restore the area that was eliminated. This frequently translates into a displacement of the red edge position towards longer wavelengths. However, Figure 5 shows that the mean standardized reflectances of tea were almost the same in each band between 18 October 2012 and 22 February 2015. The same appearance was noted for pineapple as well. For this reason, we believed that the tea LULC and tea spectral dynamics are relatively stable.

### 2.4. Generation of Other Input Variables

This study assumed that the classification accuracy of tea and non-tea LULC separation would be increased if more sub-class bands or indices were involved in the procedure. Thus, eight WorldView-2 bands treated with pan-sharpening and standardization, 64 GLCM texture indices derived from these eight bands, and eight principal components, for a total of 80 variables, were used as the input variables. In regard to the spectral variables, first, the multispectral images of the eight bands of WorldView-2 and the panchromatic images of the single band were treated with fusion using hyperspherical color spaces (HCS) [67]; this is followed by standardization which would be more convenient for the subsequent feature selection by using logistic regression (LR) analysis, which is ORIGIN 1–8 in Table 1.

In addition, PC 1–8 was generated from the analysis of ORIGIN 1–2 in the principal component analysis. In regard to the spatial characteristics, the texture indices adopted in this study consisted of mean, variance, contrast, dissimilarity, homogeneity, correlation, entropy, angular second moment in GLCM. These indices are supposed to be helpful for distinguishing the structure differences between tea and other crop fields, such as the fields of pineapple and betel palm. With regards to plant structure, the canopies of tea trees have a more compact structure compared with other crops. In addition, although the tea trees and other crops in the study area are mostly planted line by line, the canopies of tea trees usually cover most of the soil surface. Therefore, tea crop fields show smoother texture than other crop fields. The relationship between the above ecological meaning and the GLCM indices along with the formulas of each of the index are shown in Table 1. Among them, *i* is the row number, *j* is the column number; *p_i,j_* is the normalized frequencies at which two neighboring pixels separated by a constant shift occur in the WorldView-2 imagery; *N* is the number of gray levels presented in the imagery.

With the purpose of proving that the increase of “sub-categories” or “factors” could enhance the LULC classification, this study adopted four spectral distance measurements: Euclidean, divergence, transformed divergence (TD), Jefferies-Matusita (J-M), to calculate the degree of separation of the training samples of tea trees and non-tea trees (forest, building and road, water bodies and ponds and other non-tea LULC) in the spectrum. In the TD assessment, 2000 was the best categorical resolution. Basically, above 1900 represents a good categorical resolution. The closer the selected sample from the training area is to 0, the worse the categorical separation efficiency is, so training data shall be re-selected. J-M has a similar concept towards TD; the closer to 1414, the better the separability is (Table 2). The results showed that the larger the increase of sub-categorical bands or indicators is, the more effective it is to increase the separability between tea and non-tea farmland. This study subsequently investigated which sub-categorical band was the most helpful.

### 2.5. Feature Selection-LR Analysis

We applied LR analysis, a log-linear model suitable for quantitative analysis, in two different stages of this research. In feature selection stage, LR analysis was a filter to reduce the dimension of the data from 80 to less than 80. The next part we applied LR analysis was to evaluate the best LULC classifier, and LR model was one of the classifiers.

LR analysis is useful when the dependent variable is categorical (e.g., yes or no, presence or absence) and the explanatory (independent) variables are categorical, numerical, or both [68,69,70]. Generally speaking, a LR model can be expressed in the following forms [71]:

(1) $$P\left(y_{i}=1\right)={\left[1+exp\left(\alpha +x_{i}\beta \right)\right]}^{-1}$$

In Equation (1), $P\left(y_{i}=1\right)$ means the conditional probability of landslide changes; $x_{i}$ is the vector of independent variable; if $p=P\left(y_{i}=1\right)$, the above equation can be revised to:

(2) $$ln\left(\frac{p}{1-p}\right)=\alpha +\beta_{1}\;x_{1}+\beta_{2}x_{2}+\cdots +\beta_{n}x_{n}$$

In Equation (2), *p* is the occurrence probability; α is the intercept; and $\beta_{1}$, $\beta_{2}$, ... $\beta_{n}$ are the regression coefficients of the input variables $x_{1}$, $x_{2}$, ... $x_{n}$. In order to interpret the occurrence probability, one has to use the coefficients as a power to the natural log (e). The result represents the corresponding coefficient of each responding independent variable is the effect on odds (tea and non-tea occurrence), and when $\beta_{n}\;$= 0 it means that the odds will not change. In this way, we can sum up the main factors related to the land cover. Last, Cox & Snell test, Omnibus test, Hosmer and Lemeshow test and odds ratio are used for the LR model as to achieve the assessments of the explanatory power of the factors and the model fit. We applied SPSS for calculating LR here, and all input row data (.txt) were generated from original layer files (.grd) by using the conversion tools of ArcGIS software.

### 2.6. Classification Procedure

The classification in this study basically used PBIA and OBIA mainly because the high spatial resolution of pan-sharpened WorldView-2 0.5 m images may present high spectral heterogeneity of the surface features, and the LULC classification may be affected if one only includes PBIA [72]. The difference of OBIA and PBIA is that the image shall be segmented before the classification and the objects are created; the establishment of objects from image segmentation mainly uses the heterogeneity, shape and boundary of surface features to define a object. This study segmented the images with multi-scale segmentation by using the eCognition image processing software concept. During the image segmentation, the scale, shape and compactness were the three parameters which produced the most reasonable results. The reasonability defined in this study refers to: (1) the drawing of the tea tree plantation in the study area using the boundaries; and (2) the object is still complete and does not include more than one kind of LULC classification after the image segmentation. This study used ORIGIN variable layers as the image segmentation basis. The best parameters obtained were 160 in scale, 0.1 in shape and 0.5 in compactness within the study area. After obtaining the reasonable segmented results, a classification of the LULC for the segmented images follows. PBIA classifies LULC based on the value of the single pixel, yet the OBIA would assign the LULC classes with the characters of homogeneous segmented features.

In both PBIA and OBIA, the aforementioned 80 variables were a huge amount which would cause a great burden to the system operation. For this reason, LR model was applied to reduce data dimensions before putting variables into ML, LR, RF, and SVM classifiers. The area of training samples for tea and non-tea was 6.2 and 16.1 hectares separately for PBIA classifiers, and 36 and 132 segments for OBIA classifiers. The following section provides a detailed description of tea tree classification with RF and SVM.

#### 2.6.1. Classification Procedure-RF Classifier

We employed a newly developed RF algorithm as one of the LULC classifiers in this study. The main concepts of the RF algorithm are based on a non-parametric decision-tree, and it allows various kinds of inputs with different scales and types, including data sources recorded on different measurement scales [73]. This advantage allows us to use three different sets of variables involving original spectral bands and their derivative GLCM and PCA. The process of RF starts by selecting many bootstrap (with replacement) observations from the original data. The data source of a single classification tree comes from each bootstrap sample, but the predictor variables decrease with the binary partition classification tree at each node. The observations eliminated from bootstrapping at each decision-tree are called the “out-of-bag” (OOB) observations. The error rate for the entire RF classifier was generated from the comparison between the observed classification and the predicted classification for all observations derived from the out-of-bag vote tallies [74]. We thus built a separate RF model for three sets of predictors:

1. Two control parameters were set: one for the amount of the decision trees (n_tree_), while the other was for the number of characteristic parameters (m_try_);
2. We took the original training samples and then put them back with a bootstrap method; the best number and sample of decision tree *Xi* (*i* = number of iterations) were randomly collected from the original samples (dataset) *X*. Each sample stage contained about one third of *X*. The element that was not included in the sample was called OOB;
3. Every decision tree would have a complete growth without pruning. This step differs from a traditional decision tree;
4. In every iteration of the bootstrap, the prediction did not include the dependent variables within OOB, and the results generated from all the decision trees were to be averaged;
5. When all OOB data was included in the generation of decision tree, the importance of every factor could be ensured through the calculation of RMSE with increased percentage; thus, the correlations of these factors with the dependent variables could be established;
6. Last vote was to elect the classification results into tea tree or non-tea tree.

#### 2.6.2. Classification Procedure—SVM Classifier

The theoretical concept of SVM was basically developed from the optimal conditions of the hyperplane from the linear or non-linear separability. The optimal hyperplane not only correctly separates two types of samples but also has the largest class intervals. For a linearly separable binary classification, if the training data with k number of samples is represented as {*X_i_*, *y_i_*}, *i* = 1, …, *k* where $X\in R^{N}$ is an *N*-dimensional space and y∈{+1,−1} is a class label with two classes, the SVM classifier can be created by a hyperplane given by:

(3) $$\left\{ \begin{array}{l} (W\cdot X_{i})+b=+1\\ (W\cdot X_{i})+b=-1 \end{array}\right.$$

where *W* is a vector perpendicular to the linear hyperplane and *b* is a scalar showing the offset of the discriminating hyperplane from the origin. For the two classes *y*, two hyperplanes can be used to discriminate the data points in the respective classes. The two hyperplanes can be expressed as:

(*W* · *X_i_*) + *b* ≥ +1 for all y = + 1, *i.e.*, class 1, and (4)

(*W* · *X_i_*) + *b* ≤ −1 for all y = − 1, *i.e.*, class 2 (5)

For a nonlinear classification, the input data is mapped onto a higher dimensional feature space using a non-linear mapping function. The classification is then carried out in the mapped space rather than the input feature space. In order to increase the computational efficiency, a kernel-trick is usually used to definite kernel function. In this study, we chose the kernel function contributing to the best tea and non-tea LULC classification from the four basic options: linear, polynomial, radial and sigmoid. Besides, two crucial parameters, cost of constraints violation (C) and sigma (σ), were also considered to create the best SVM classifier. C accounts for the over-fitting of the model while σ decides the shape of the hyperplane [65].

### 2.7. Accuracy Assessment

For accuracy assessment of PBIA results, we converted the ground reference data from vector to grid file format with a cell size of 0.5 m (*i.e.*, the same cell size as the spatial resolution of pan-sharpened WorldView-2 images) and calculated the areas classified correctly and incorrectly, while for OBIA we calculated the number of segmented objects rather than the areas with correct and incorrect classification results. Indices including producer’s accuracy (PA), user’s accuracy (UA), overall accuracy (OA) and kappa index of agreement (KIA) were then applied to evaluate the PBIA and OBIA results. The KIA measures the difference between the actual agreement and the chance agreement in the error matrix [75,76]. The KIA is computed as:

(6) $$KIA=\frac{N\sum_{i=1}^{r}x_{ii}-\sum_{i=1}^{r}x_{i+}\cdot x_{+i}}{N^{2}-\sum_{i=1}^{r}x_{i+}\cdot x_{+i}}$$

where *r* is the number of rows in the error matrix; *x_ii_* is the number of observations in row *i* and column *i* (on the major diagonal); *x_i+_* is the total of observations in row *i*; *x_+i_* is the total of observations in column *i*; *N* is the total number of observations included in the matrix.

To assess if the statistical difference between the results of PBIA and OBIA exists, McNemar’s test based on the standardized normal test statistic was used in this study. The difference in accuracy between two approaches is statistically significant if |*z*| > 1.96, and the sign of *z* indicates which the more accurate approach is [77,78]. The *z*-score is computed as:

(7) $$z=\frac{f_{GP}-f_{PG}}{\sqrt{f_{GP}+f_{PG}}}$$

where *f_GP_* indicates the number of samples correctly classified by OBIA and incorrectly classified by PBIA and *f_PG_* indicates the number of samples correctly classified by PBIA and incorrectly classified by OBIA.

## 3. Results

In this section, we describe the feature selection results, followed by the classification ones. When discussing the results, we mostly focus on the tea cultivation area.

### 3.1. Feature Selection

Although both PBIA and OBIA had 80 input variables, yet their “basic-unit values” were different; thus, LR model filter shall be carried out separately as to select the best variables for pixel-based and object-based approaches.

In terms of feature selection in a pixel-based approach, the eight multispectral bands, the eight principal components and the 64 GLCM textures were used to choose the significant impact variables in the LR analysis. The analytical result obtained with Cox & Snell R square test was 0.481. The *p* value of the Omnibus test was 0.000, while *p* < 0.01 meant the explained variance of independent variables was significantly greater than the variance of the unexplained ones. This also represented that there was at least one independent variable which can effectively explain and predict the dependent variable. The *p*-value of Hosmer and Lemeshow test was 0.511. The insignificant *p*-value here meant LR model was indicated of good fit. With the 34 significant variables listed in Table 3, the best classification accuracy (87.5%) gained from the training data can be achieved. 

Among them, the 1st to the 21th represents the texture variables. MEAN2 means the MEAN texture of the second band in WorldView-2 fusion image. The others may also be induced in the same way. From the results, the ENTROPY and SECOND MOMENT textures in each band were both considered as not significant, so they were both removed from the classifiers. MEAN7 has the highest B coefficient among all texture variables and this implies that it is the most beneficial index to differentiate tea from non-tea LULC. PC1 means the first main component. Except for PC6, the rest of the main components were significant; ORIGIN, which was the original band of WorldView-2 fusion image, was not significant in the first and fifth band of the model; thus, only the other six bands were kept. The variables from the aforementioned Table 3 were the input variables of the four pixel-based classifiers. In terms of feature selection step in OBIA, the Cox & Snell R square value of the analytical results was 0.547, meaning the intensity of correlation and the predictive power of the input variables and dependent variables are both good. The result of the Omnibus test was the value of *p* = 0.000, meaning that the explained variance was significantly greater than the unexplained variance, and among the input variables, at least one variable can effectively explain and predict the dependent variable. The *p*-value of Hosmer and Lemeshow test was 0.977. It meant LR model was indicated of good fit. The six significant variables listed in Table 4 can be used to achieve the best classification accuracy (94.7%) from the training data.

The difference between OBIA and PBIA consisted in the variables which were related to spectral characteristics, such as MEAN, ORIGIN and PC, and they were not considered as significant; however, the similarity among them is that CONTRAST, DISSIMI and CORREL were considered as significant. This means when the classification was carried out with OBIA, the spatial characteristics would have more referential meaning than the spectral characteristics. This feature could avoid the high spectral heterogeneity phenomenon in high spatial resolution images [72]. The variables in the aforementioned Table 4 were taken as the input variables of the four object-based classifiers.

### 3.2. Classification with the Selected Input Variables

The following two sub-sections demonstrated the results generated from ML, LR, RF and SVM classifiers with the use of 34 and six selected variables for the pixel-based and object-based approaches respectively.

#### 3.2.1. Pixel-Based Results

**Figure 6:**
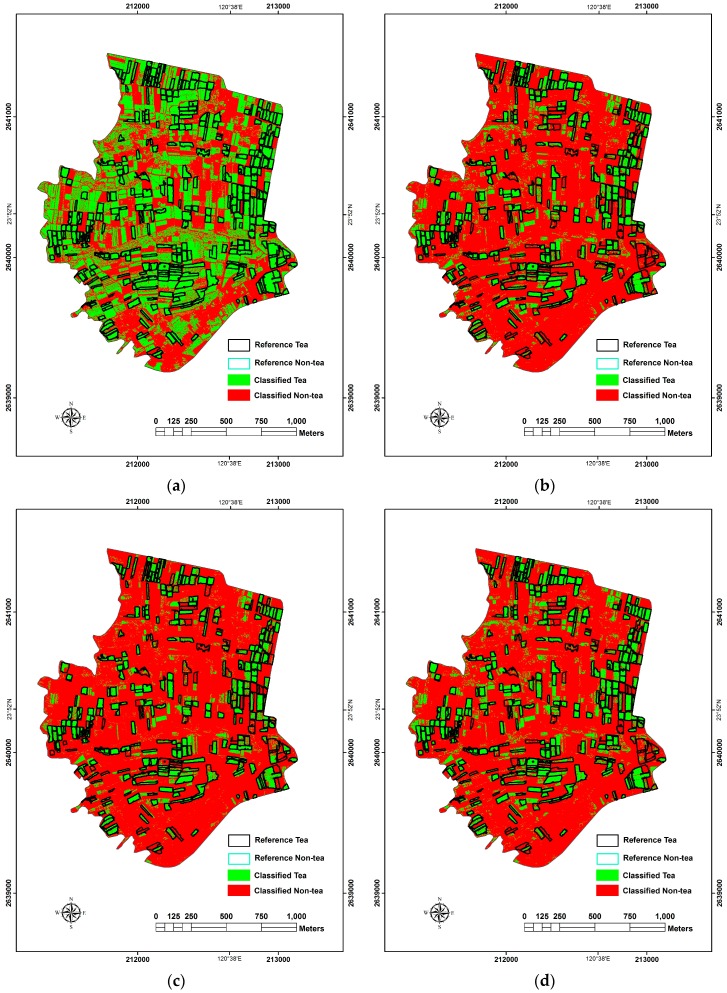
The results of PBIA classification. (**a**) ML; (**b**) LR; (**c**) RF; (**d**) SVM.

The study evaluated the classification accuracies of ML, LR, RF and SVM classifiers seperately in PBIA. First, in the LR analysis, we used Equation (2) and the B coefficient in Table 3 to derive the occurrence probability *p*; pixels with the value of *p* < 0.5 were assigned as the class of tea while non-tea if *p* ≥ 0.5. RF classifier takes the best n_tree_ = 500 and m_try_ = 11 as the final parameter setting. SVM classifier obtained the best classification results with radial kernel function and gamma value of 0.1. The best results obtained with the use of the aforementioned classifiers were shown in Table 5 and Figure 6. 

In terms of classification accuracy, SVM was the highest (OA = 84.70% and KIA = 0.690) followed by RF analysis, ML and LR. In terms of McNemar’s test, the accuracy of SVM which was the best in classification was significantly higher than the second best RF (|*z*| = 35.961), while LR was significantly the lowest (with |*z*| = 72.109 compared with ML).

For more detailed information about the PA and UA values of each classifier, we found that the PA value of ML classifier was much higher than others (Figure 6a) since over 50% of study area was interpreted as tea tree LULC. It obviously reduced the omission error in tea tree classification (*i.e.*, 1—PA); however, it also caused several non-tea areas to be commissioned as the classof tea and decreased the UA of the class of tea and the PA of class of non-tea.

#### 3.2.2. Object-Based Results

**Figure 7:**
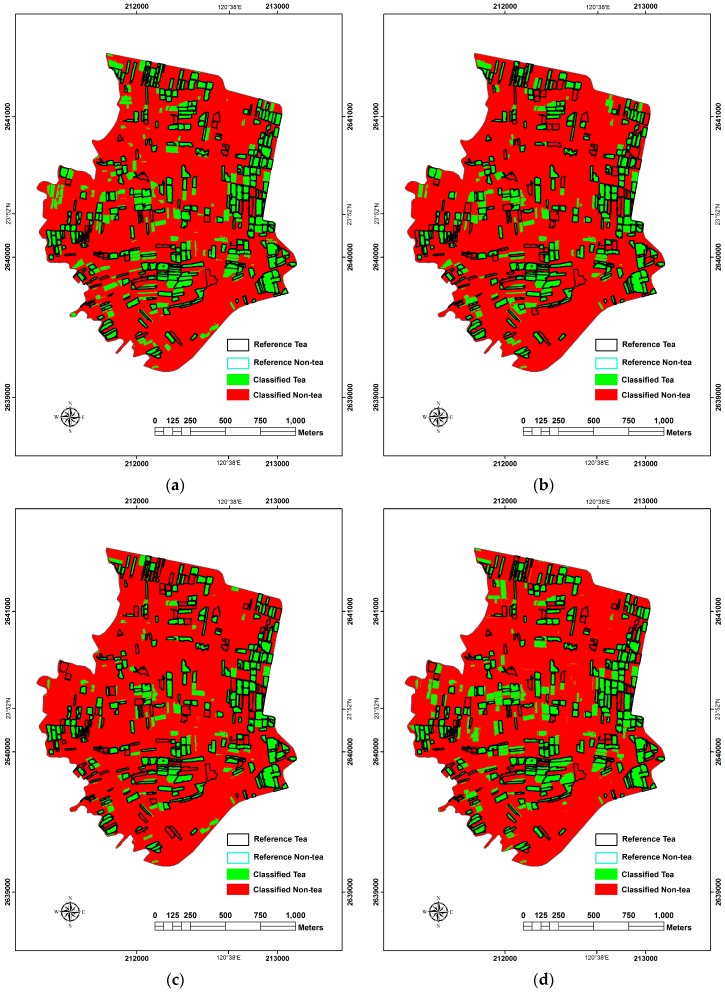
The results of OBIA classification. (**a**) ML; (**b**) LR; (**c**) RF; (**d**) SVM.

In the LR analysis, the same as the pixel-based approach, objects with the value of *p* < 0.5 were assigned as the class of tea while they were non-tea. RF classifier obtained the best classification results when n_tree_ = 500 and m_try_ = 3. SVM classifier obtained the best classification results with radial kernel function and gamma values of 0.1. The best results gained with the aforementioned classifiers are shown in Table 6 and Figure 7. 

In terms of the accuracy of classification, ML was the one with the highest member in OBIA. OA and KIA may both reach to 96.04% and 0.887. LR, SVM analysis and RF followed. The one with the lowest accuracy was RF in OA and KIA with 91.20% and 0.733 respectively. The results of McNemar’s test showed that ML was with the best classification accuracy which was significantly higher than the one of the second best LR (|*z*| = 138.082), while the accuracy of SVM analysis was significantly higher than RF (|*z*| = 38.051). Evaluating the details of PA and UA of the classifiers, the PA of tea tree with ML was higher than the other classifiers, which was the main reason for it to give the best overall classification results. The PA of tea tree and the UA of non-tea tree with RF were not high, meaning that many tea tree areas were classified as non-tea tree areas, which was the main reason for it to be the worse results among the classifiers.

#### 3.2.3. Overall Comparison

Using the same classifier to compare the results obtained with the use of PBIA and OBIA, the classification accuracy of OBIA among ML and LR analysis classifiers was higher, while the one among the results gained with the use of PBIA in SVM and RF was higher. Although the result of McNemar’s test indicated that both results had significant differences, the significant values |z| of ML and LR analysis classifiers were larger than the ones of RF and SVM. Overall, it turned out that the results obtained with the use of OBIA were better than the results obtained with PBIA in both OA and KIA (Table 7).

What is worth attention is that the accuracy of OBIA + ML was not only higher than that of PBIA + ML but also the best among all the results obtained with PBIA and OBIA. In addition, if we take a look at the ML results of the two approaches, the PA of tea tree LULC is the highest among all the classifiers mainly because the ML, whether among PBIA or OBIA results, tended to classify more areas as tea tree LULC as to avoid the error caused by omission. The performance of all classifiers of PBIA and OBIA approaches were unstable while the OA varied a lot between 82.97% and 96.04%. However, the results of RF in this study showed a difference from those of the previous studies for other LULC classification which showed the highest OA result than other classifiers [65,79]. It was speculated that perhaps in the experiment design of this study, only two categories—tea and non-tea LULC were provided—tea tree and non-tea tree LULC so that RF cannot show its advantage in classification.

## 4. Discussion

In the feature selection stage, adding variables such as GLCM layer would increase the interpretation capability of land-use/land cover, but it requires a lot of time and efforts. This study tried to find the most useful variables to be added here so we can promote the efficiency of interpretation with lowest cost. As mentioned in Figure 2, the feature selection stage based on LR model delineated 34 useful variables in PBIA and 6 GLCM variables in OBIA. All selected variables were significant related factor for LR function (*p* < 0.05, and B coefficient can be considered as the weights of importance). It means that we only need to input 34 variables in PBIA classifier, and six variables in the OBIA classifier. We think it is important for saving money if the government (e.g., Council of Agriculture) needs to lower the cost of crop cultivation investigation.

**Figure 8:**
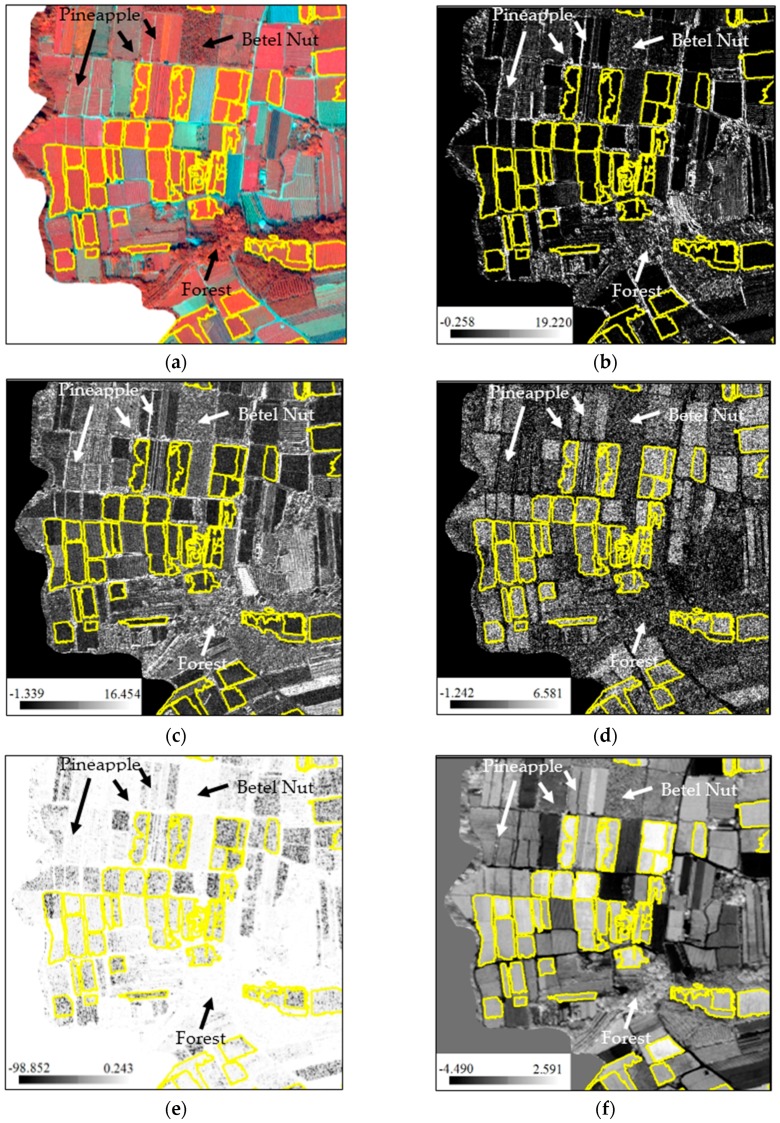
Signature comparison of tea, betel nut, pineapple and forest fields using input variables with higher B coefficient. (**a**) is the original image displayed with band 7, 3 and 2; while (**b**–**f**) are standardized CONTRAST6, DISSIMI6, HOMO2, CORREL6 and PC2 respectively. The yellow boundaries delineate the tea crop fields.

Previous studies have already discussed the use of four newly available spectral bands (coastal, green, yellow, red edge) for the classification of the tree species [39,80]. In this research, the results of feature selection using LR analysis showed that the influence of band 6 (red edge) and band 4 of its derivative texture indices were significant to the tea classification. Figure 8 shows the signature comparison between tea, betel nut, pineapple and forest fields using input variables with different band combination. Obviously, the visual differences became clear when standardized CONTRAST6, DISSIMI6, HOMO2, CORREL6 and PC2 were included in classification process. This means red edge spectrum and some texture indices were useful in distinguishing between tea and non-tea LULC. It also supports the conclusions of the previous studies concerning vegetation classification [39,81]. The superiority of the red edge spectrum indicates that it can reflect the chlorophyll concentration and the physiological status of crops [82]; and thus it can also be helpful for land cover classification [81]. Besides, principal component transformation is commonly considered a good method to reduce data dimension and to extract useful information. After the processing of 8-band WorldView-2 imagery, the most abundant information was transformed into the first PC (PC1) and the information decreases gradually to the last PC (PC8). According to the LR analysis results, however, PC2 to PC8 except PC6 were still helpful for tea and non-tea LULC discrimination. This implies that every component may have to be thought about when conducting binary classification. LR analysis also indicates the importance of each variable. Taking tea, betel nut, pineapple fields and forest as the examples, Figure 8 shows that the input variables with higher absolute B coefficient values differentiate the LULC above more than those with lower B coefficients.

As for textural information, LR analysis in feature selection showed that GLCM MEAN and DISSIMI were the two most dominant variables for the tea LULC classification. GLCM MEAN represented the local mean value of the processing window and had high relation with the original spectral information. The processing window also called moving window, is the basic unit for calculating grid layer features. The value of each pixel is calculated by shifting the window one pixel at a time until all pixels are calculated. The size of moving window was 3 × 3 pixels in this study. B coefficients of the MEAN which were higher than those of other variables suggested that the spectral information was more useful than the structural characteristics of WorldView-2 imagery. Nevertheless, the dissimilarity measures, which were based on empirical estimates of the feature distribution, were also critical for enhancing the structural difference between tea and non-tea LULC.

**Figure 9:**
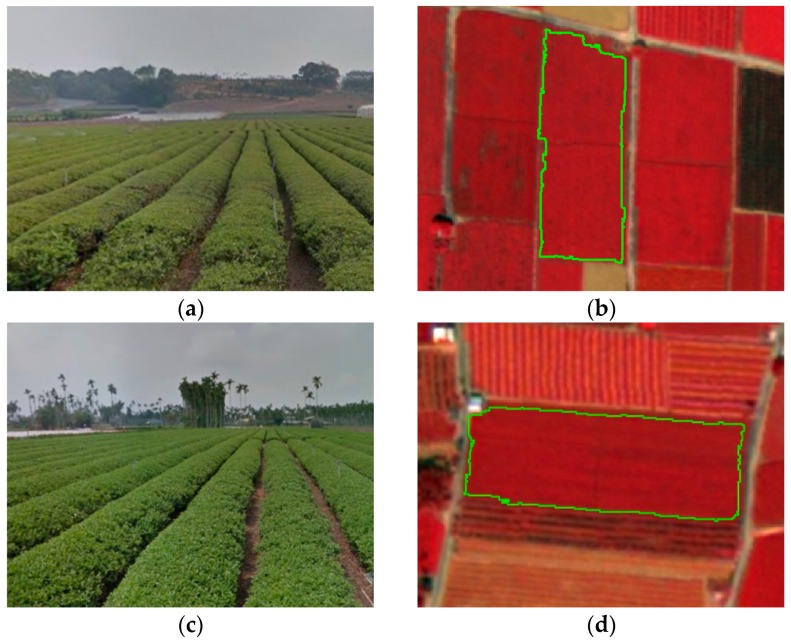
Comparative images of the photograph of the tea tree field (left row) and WorldView-2 (right row). (**a**) is the photograph captured in 120.6341°E, 23.8684°N in 4 March 2015 while (**b**) is its corresponding WorldView-2 image captured in 22 February 2015; (**c**) is the photograph captured in 120.6292°E, 23.8615°N in 5 March 2015 while (**d**) is its corresponding WorldView-2 image captured in 22 February 2015.

Though previous studies seldom used MEAN and DISSIMI for tea classification, these two measures had been proven effective in improving classification accuracy for agricultural and forest areas [65,83] and the idea also indirectly support the findings of this study. The tea trees in the study area were planted in rows with totally different characteristics from those of the crops observed on the ground in structure and arrangement. However, in Table 3, the overall spectral variables of B coefficient are still larger than the structural variables when using pixel-approach. This means that in pixel-based classification, the spectral variables were more effective in distinguishing the differences of the ground coverage of tea trees and non-tea trees comparing to the structural variables. This could be explained with the two examples in Figure 9. Even in the pan-sharpened WorldView-2 imagery with 0.5-m high spatial resolution, there are not all characteristics of the line by line formation could be clearly presented due to the narrow interval between tea trees. For this reason, it seems to be much harder for the structural variables to play to their strengths.

As for the issues for reducing data dimensions and the number of input variables, the comparative analysis between PBIA and OBIA in this study demonstrated a significant difference that using OBIA was more effective and sensitive in conducting variable filtering process than using PBIA. The strength of OBIA was attributed to the less amount of samples and the reduction of spectral heterogeneity within each variable layer when producing objects from pixels. Taking this study for example, it means that people could spend less time generating input variables and achieve satisfactory results compared to using PBIA if OBIA is applied. However, it is worth noting that OBIA demands more computational resources, requires specialized software (*i.e.*, eCognition Suite) and is hard to applied in a large study area even the number of objects is much less than the total number of pixels. The above mentioned factors often set limitations on the use of OBIA.

One parametric classifier (ML) and three non-parametric classifiers (LR, SVM and RF) were applied in this study. According to the accuracy assessment of PBIA and OBIA approaches, almost all OA values were found to exceed 82.97%. The use of OBIA + ML obviously got the best OA value among OBIA approaches, but the use of PBIA+ML did not. The main reason that the overall accuracy of ML classifier was not as good as non-parametric approach (SVM or RF) in PBIA comes from the bimodal data distribution of tea-farm training samples due to the alternate permutation of tea plants and corridors. The special arrangement might lead tea farms divided into two LULC types in PBIA when ML classier was applied. However, in OBIA, the basic unit was polygon that contained many WorldView-2 image pixels, the average treatment effect within tea farm training samples would decrease the difference of every basic unit, eliminate the spectral heterogeneity within every tea LULC patches, and lead sample distribution more like normal distribution. The ML classifier performs well for normally distributed data, this is why the classification result of the use of OBIA + ML was much better than PBIA + ML.

Minimum distance classifier has been commonly used to classify remote sensing imagery [84,85]. This classifier works well when the distance between means is large compared to the spread (or randomness) of each class with respect to its mean; moveover, it is very good approach dealing with non-normal distribution data. However, this study did not consider this classifier as one of the methods in PBIA and OBIA approaches. The reason is that the non-tea class spreads much wider in the feature space and thus the distance between the means of non-tea and tea classes is not large compared to the spread of non-tea class. In this case, if there is an unclassed sample situating near the boundary of non-tea and tea classes in the feature space, the classification result may not be ideal. As for the ML classifier, the occurrence frequencies of non-tea and tea classes are used to aid the classification process. Without this frequency information, the minimum distance classifier can yield biased classifications. Although salt and pepper effect may influence the distribution normality of the data, OBIA approach can decrease the possibility of salt and pepper effect. Therefore, we think ML classifier still has the superiority especially in OBIA approach.

As for SVM classifier, its superior classifying effects toward high-dimensional feature space, capability on processing non-linear data, characteristics to avoid over fitting and under fitting, and the stability to decrease the salt and pepper effect made it the best classifier of PBIA. However, it was not the best algorithm of OBIA. We inferred the most possible reasons: (1) the OBIA image segmentation stage in this study made the sample data look like a case of normal distribution, decreased the possibility of salt and pepper effect, and promoted the effects of ML algorithm; (2) OBIA image segmentation was possible to combine tea farms and pineapple farms together due to the line by line arrangements of tea trees and pineapples with unfixed interval. Although the advantages of SVM with soft margin were not presented in the application of OBIA, the OA and Kappa values were still good.

As for the LULC monitoring frequency, compared with many herbaceous crops, the monitoring task on tea trees with high spatial resolution commercial satellite imagery is much easier because of the long-lived perennial characteristics of tea tree. The relatively stable life cycle of tea production in Taiwan often starts on the 3rd year after the planting of saplings, and then there is going to be one to two harvests every season for at least 30 years. Therefore, people do not need to collect a series of period images as frequently as in the monitoring on herbaceous crops. Nevertheless, it is worth mentioning that this study still has some limitations. The first one is the study area of this research was limited to a flat and gentle sloping area, yet some tea farms are in the high mountains where the terrain effect would certainly have an influence due to the shadow-effect of satellite images. For this reason, we suggest the users to apply our workflow in flat or gentle slope areas. Besides, at some places, physiological changes among non-tea plants might influence the spectral reflection of satellite images, so we considered that our workflow is suitable for the use in sub-tropical places where most plants are evergreen.

After all, in order to achieve tea production prediction or evaluation of high efficiency and low costs, we suggest the governments apply the methodology used in this study as a standardized framework, while field surveys and re-investigation with manpower resources should be the backup option for calibration or supplementary once people want to totally eliminate the commission and omission errors. The classification method in this study can be further applied in two different ways. The first one is of benefit to countries with the largest tea planting areas (e.g., China, India, Kenya, Sri Lanka, and Vietnam). Besides, it can also be applied in other crop classification studies such as banana, pineapple, sugar cane, and watermelon with particular type of plantation and farming arrangement.

## 5. Conclusions

Traditional field surveys or orthogonal photograph interpretation for the monitoring of sensitive crops are time-consuming and expensive. The assistance of high spatial and spectral resolution imagery has been proven useful for mapping several crops and helpful in agriculture land management and sustainable agricultural production. However, there is a gap in the research on mapping tea LULC in intensive and heterogeneous agriculture areas due to the spectral and spatial resolution restrictions. This study investigated the WorldView-2 imagery characteristics and the most applied significant variables and classification approaches used to develop a stepwise classification framework for tea tree mapping in Taiwan. The results show it is not enough for tea and non-tea crop classification if we only apply the original approach of eight MS bands of WorldView-2. Advanced variables such as GLCM texture indices are necessary. It is evident that logistic regression analysis in this study was helpful in feature selection, and the OBIA approach filtered more insignificant variables than the PBIA approach. In other words, the OBIA approach costs less but generated additional variables.

As for higher accuracy of tea tree classification, one parametric classifier (ML) and three non-parametric classifiers (LR, SVM and RF) were applied. The obtained data showed that all classifiers achieved overall accuracies ranging from 82.97% to 96.04%. Parametric ML showed significantly higher accuracy in OBIA while the lower accuracy of PBIA was due to the different spectral heterogeneity in the tea tree samples. We suggest that the OBIA+ML approach would offer the best combination of efficiency and stabilization in tea tree classification. Overall, the applicability of remote sensing imagery for the evaluation, and monitoring of tea trees was well confirmed.

## Figures and Tables

**Table 1:** Description of the input variables used for the classification.

Variable	Symbol	Formula	Description
Bands 1–8	ORIGIN 1–8	--	Pan-sharpened and standardized 8 WorldView-2 bands.
GLCM Mean 1–8	MEAN 1–8	$\sum_{i,j=0}^{N-1}\frac{P_{i,j}}{N^{2}}$	The local mean grey level value in a given area. This measure helps distinguish the spectral difference between tea and other crop fields.
GLCM Variance 1–8	VARIANCE 1–8	$\sum_{i,j=0}^{N-1}p_{i,j}{\left(i-\mu_{i}\right)}^{2}$	A measure of heterogeneity. Variance increases when the grey level values differ from their mean. Tea crop fields usually show lower variance compared with other crops.
GLCM Contrast 1–8	CONTRAST 1–8	$\sum_{i,j=0}^{N-1}p_{i,j}{\left(i-j\right)}^{2}$	Measures the linear dependency of grey levels of neighboring pixels. High contrast represents heavy textures. Tea crop fields usually show lower contrast compared with other crops.
GLCM Dissimilarity 1–8	DISSIMI 1–8	$\sum_{i,j=0}^{N-1}p_{i,j}\left|i-j\right|$	Defines the variation of grey level pairs in an image. It is the closest to Contrast with a difference in the weight. Contrast will always give slightly higher values than Dissimilarity.
GLCM Homogeneity 1–8	HOMO 1–8	$\sum_{i,j=0}^{N-1}\frac{P_{i,j}}{1+{\left(i-j\right)}^{2}}$	Measures the level of variation in a given area. High homogeneity refers to textures that contain ideal repetitive structures. Tea crop fields usually show higher homogeneity compared with other crops.
GLCM Correlation 1–8	CORREL 1–8	$\sum_{i,j=0}^{N-1}p_{i,j}\left(\frac{\left(i-\mu_{i}\right)\left(j-\mu_{j}\right)}{\sigma_{i}\sigma_{j}}\right)$	A measure of grey level linear dependence between the pixels at the specified positions relative to each other. Tea crop fields usually show higher correlation compared with other crops.
GLCM Entropy 1–8	ENTROPY 1–8	$\sum_{i,j=0}^{N-1}p_{i,j}\left(-\ln p_{i,j}\right)$	Measures the level of chaos in a given area. A completely random distribution would have very high entropy because it represents chaos. Solid tone image would have an entropy value of 0. Tea crop fields usually show lower entropy compared with other crops.
GLCM Angular second moment 1–8	ASM 1–8	$\sum_{i,j=0}^{N-1}p_{i,j}^{2}$	Measures the textural uniformity that is pixel pair repetitions. High ASM values occur when the grey level distribution is constant or periodic. Tea crop fields usually show higher ASM values compared with other crops.
Principal Component 1–8	PC 1–8	--	Principal components derived from ORIGIN 1–8.

**Table 2:** Various spectral distance measurements between tea and non-tea (including non-tea LULC, forest, water and ponds, buildings and roads) training area.

Measurement	Distance
Euclidean	4.05
Divergence	14,288.60
TD	2000.00
J-M	1414.00

**Table 3:** Feature selection results of PBIA with LR model.

Variable	B Coefficient	S.E.	Wald	*p*-Value	Odds Ratio
MEAN2	−2.702	0.072	1401.956	0.000	0.067
MEAN3	2.199	0.084	686.504	0.000	9.020
MEAN4	1.528	0.072	448.174	0.000	4.609
MEAN6	−1.519	0.068	493.346	0.000	0.219
MEAN7	3.963	0.075	2823.246	0.000	52.629
MEAN8	−3.176	0.081	1526.896	0.000	0.042
VARIANCE3	−0.870	0.070	154.513	0.000	0.419
VARIANCE6	1.280	0.050	646.022	0.000	3.597
CONTRAST1	−0.604	0.052	134.969	0.000	0.546
CONTRAST6	1.903	0.063	900.445	0.000	6.704
CONTRAST8	0.129	0.029	19.601	0.000	1.138
DISSIMI3	−0.500	0.039	164.181	0.000	0.606
DISSIMI5	−0.103	0.031	11.042	0.001	0.902
DISSIMI6	0.658	0.022	899.772	0.000	1.931
DISSIMI7	0.364	0.027	187.321	0.000	1.440
DISSIMI8	−0.569	0.029	388.366	0.000	0.566
HOMO1	0.043	0.005	86.851	0.000	1.044
HOMO2	0.069	0.004	250.479	0.000	1.071
CORREL2	−0.025	0.003	74.088	0.000	0.975
CORREL3	−0.036	0.003	110.817	0.000	0.965
CORREL6	−0.050	0.004	194.260	0.000	0.951
PC1	−3.898	0.137	812.674	0.000	0.020
PC2	−4.083	0.116	1230.779	0.000	0.017
PC3	−3.216	0.126	652.325	0.000	0.040
PC4	−0.643	0.044	211.023	0.000	0.526
PC5	0.352	0.025	193.298	0.000	1.423
PC7	−0.180	0.011	245.752	0.000	0.835
PC8	0.221	0.015	211.578	0.000	1.247
ORIGIN2	−2.792	0.118	556.104	0.000	0.061
ORIGIN3	1.685	0.099	287.170	0.000	5.390
ORIGIN4	3.604	0.102	1256.850	0.000	36.740
ORIGIN6	−2.679	0.101	706.748	0.000	0.069
ORIGIN7	2.683	0.135	392.887	0.000	14.635
ORIGIN8	−1.922	0.135	201.614	0.000	0.146
Intercept	3.826	0.012	95,806.689	0.000	45.866

**Table 4:** Feature selection results of OBIA using LR model.

Variable	B Coefficient	S.E.	Wald	*p*-Value	Odds Ratio
CONTRAST3	0.024	0.007	11.481	0.001	1.024
DISSIMI2	−1.439	0.467	9.473	0.002	0.237
DISSIMI5	−0.475	0.151	9.935	0.002	0.622
CORREL2	−65.267	20.660	9.980	0.002	0.000
CORREL3	139.379	39.486	12.460	0.000	3.402 × 10^60^
CORREL6	−30.690	8.535	12.929	0.000	0.000
Intercept	−8.239	20.695	0.158	0.691	0.000

**Table 5:** Error matrix for pixel-based classification with 34 selected variables; the unit is pixel number.

**(a) Pixel-Based ML**				
		**Reference**		
		**Tea**	**Non-Tea**	**UA**
**Classified**	Tea	1,804,612	577,088	75.77%
	Non-tea	110,352	1,553,379	93.37%
	PA	94.24%	72.91%	
OA = 83.01% Kappa = 0.663				
**(b) Pixel-Based Logistic**				
		**Reference**		
		**Tea**	**Non-Tea**	**UA**
**Classified**	Tea	1,353,349	127,503	91.39%
	Non-tea	561,626	2,002,973	78.10%
	PA	70.67%	94.02%	
OA = 82.97% Kappa = 0.654				
**(c) Pixel-Based RF**				
		**Reference**		
		**Tea**	**Non-Tea**	**UA**
**Classified**	Tea	1,336,860	72,876	94.83%
	Non-tea	578,104	205,7591	78.07%
	PA	69.81%	96.58%	
OA = 83.91% Kappa = 0.673				
**(d) Pixel-Based SVM**				
		**Reference**		
		**Tea**	**Non-Tea**	**UA**
**Classified**	Tea	1,431,223	135,319	91.36%
	Non-tea	483,741	1,995,148	80.49%
	PA	74.74%	93.65%	
OA = 84.70% Kappa = 0.690				

**Table 6:** Error matrix for object-based classification with six selected variables; the unit is object number.

**(a) Object-Based ML**				
		**Reference**		
		**Tea**	**Non-Tea**	**UA**
**Classified**	Tea	234	11	95.51%
	Non-tea	34	858	96.19%
	PA	87.31%	98.73%	
OA = 96.04% Kappa = 0.887				
**(b) Object-Based Logisitic**				
		**Reference**		
		**Tea**	**Non-Tea**	**UA**
**Classified**	Tea	203	6	97.13%
	Non-tea	65	863	93.00%
	PA	75.75%	99.31%	
OA = 93.76% Kappa = 0.812				
**(c) Object-Based RF**				
		**Reference**		
		**Tea**	**Non-Tea**	**UA**
**Classified**	Tea	185	17	91.58%
	Non-tea	83	852	91.12%
	PA	69.03%	98.04%	
OA = 91.20% Kappa = 0.733				
**(d) Object-Based SVM**				
		**Reference**		
		**Tea**	**Non-Tea**	**UA**
**Classified**	Tea	212	26	89.08%
	Non-tea	56	843	93.77%
	PA	79.10%	97.01%	
OA = 92.79% Kappa = 0.792				

**Table 7:** Comparison of the accuracy assessment of pixel- and object-based approaches.

		Tea		Non-Tea		OA.	KIA
		PA	UA	PA	UA		
Pixel-based	ML	94.24%	75.77%	72.91%	93.37%	83.01%	0.663
	Logistic	70.67%	91.39%	94.02%	78.10%	82.97%	0.654
	RF	69.81%	94.83%	96.58%	78.07%	83.91%	0.673
	SVM	74.74%	91.36%	93.65%	80.49%	84.70%	0.690
Object-based	ML	87.31%	95.51%	98.73%	96.19%	96.04%	0.887
	Logistic	75.75%	97.13%	99.31%	93.00%	93.76%	0.812
	RF	69.03%	91.58%	98.04%	91.12%	91.20%	0.733
	SVM	79.10%	89.08%	97.01%	93.77%	92.79%	0.792

## References

[1] Barlow K.M., Christy B.P., O’Leary G.J., Riffkin P.A., Nuttall J.G. (2015). Simulating the impact of extreme heat and frost events on wheat crop production: A review. Filed Crop. Res..

[2] Kou N., Zhao F. (2011). Effect of multiple-feedstock strategy on the economic and environmental performance of thermochemical ethanol production under extreme weather conditions. Biomass Bioenergy.

[3] Leblois A., Quirion P., Sultan B. (2014). Price *vs.* weather shock hedging for cash crops: Ex ante evaluation for cotton producers in Cameroon. Ecol. Econ..

[4] Van Bussel L.G.J., Müller C., van Keulen H., Ewert F., Leffelaar P.A. (2011). The effect of temporal aggregation of weather input data on crop growth models’ results. Agric. For. Meteorol..

[5] Van Oort P.A.J., Timmermans B.G.H., Meinke H., Van Ittersum M.K. (2012). Key weather extremes affecting potato production in The Netherlands. Eur. J. Agron..

[6] Gbegbelegbe S., Chung U., Shiferaw B., Msangi S., Tesfaye K. (2014). Quantifying the impact of weather extremes on global food security: A spatial bio-economic approach. Weather Clim. Extrem..

[7] Huang J., Jiang J., Wang J., Hou L. (2014). Crop Diversification in Coping with Extreme Weather Events in China. J. Integr. Agric..

[8] Iizumi T., Ramankutty N. (2015). How do weather and climate influence cropping area and intensity?. Glob. Food Sec..

[9] Silva J.A., Matyas C.J., Cunguara B. (2015). Regional inequality and polarization in the context of concurrent extreme weather and economic shocks. Appl. Geogr..

[10] Van Wart J., Grassini P., Yang H., Claessens L., Jarvis A., Cassman K.G. (2015). Creating long-term weather data from thin air for crop simulation modeling. Agric. For. Meteorol..

[11] Zhang W., Zheng C., Song Z., Deng A., He Z. (2014). Farming systems in China: Innovations for sustainable crop production. Crop Physiology: Applications for Genetic Improvement and Agronomy.

[12] Wijeratne M.A. (1996). Vulnerability of Sri Lanka tea production to global climate change. Climate Change Vulnerability and Adaptation in Asia and the Pacific.

[13] Azapagic A., Bore J., Cheserek B., Kamunya S., Elbehri A. (2016). The global warming potential of production and consumption of Kenyan tea. J. Clean. Prod..

[14] Smit B., Cai Y. (1996). Climate change and agriculture in China. Glob. Environ. Chang..

[15] Liu Z.-W., Wu Z.-J., Li X.-H., Huang Y., Li H., Wang Y.-X., Zhuang J. (2016). Identification, classification, and expression profiles of heat shock transcription factors in tea plant (Camellia sinensis) under temperature stress. Gene.

[16] Croce K.-A. (2015). Latino(a) and Burmese elementary school students reading scientific informational texts: The interrelationship of the language of the texts, students’ talk, and conceptual change theory. Linguist. Educ..

[17] Easterling W.E. (1996). Adapting North American agriculture to climate change in review. Agric. For. Meteorol..

[18] Hansen M.C., Egorov A., Potapov P.V., Stehman S.V., Tyukavina A., Turubanova S.A., Roy D.P., Goetz S.J., Loveland T.R., Ju J. (2014). Monitoring conterminous United States (CONUS) land cover change with Web-Enabled Landsat Data (WELD). Remote Sens. Environ..

[19] McCullum C., Benbrook C., Knowles L., Roberts S., Schryver T. (2003). Application of Modern Biotechnology to Food and Agriculture: Food Systems Perspective. J. Nutr. Educ. Behav..

[20] Gollapalli M., Li X., Wood I. (2013). Automated discovery of multi-faceted ontologies for accurate query answering and future semantic reasoning. Data Knowl. Eng..

[21] Baker E.W., Niederman F. (2014). Integrating the IS functions after mergers and acquisitions: Analyzing business-IT alignment. J. Strateg. Inf. Syst..

[22] Milenov P., Vassilev V., Vassileva A., Radkov R., Samoungi V., Dimitrov Z., Vichev N. (2014). Monitoring of the risk of farmland abandonment as an efficient tool to assess the environmental and socio-economic impact of the Common Agriculture Policy. Int. J. Appl. Earth Obs. Geoinf..

[23] Wilson B.T., Lister A.J., Riemann R.I. (2012). A nearest-neighbor imputation approach to mapping tree species over large areas using forest inventory plots and moderate resolution raster data. For. Ecol. Manag..

[24] Vegas Galdos F., Álvarez C., García A., Revilla J.A. (2012). Estimated distributed rainfall interception using a simple conceptual model and Moderate Resolution Imaging Spectroradiometer (MODIS). J. Hydrol..

[25] Sheeren D., Bonthoux S., Balent G. (2014). Modeling bird communities using unclassified remote sensing imagery: Effects of the spatial resolution and data period. Ecol. Indic..

[26] Li W., Li H., Zhao L. (2011). Estimating Rice Yield by HJ-1A Satellite Images. Rice Sci..

[27] Setiawan Y., Lubis M.I., Yusuf S.M., Prasetyo L.B. (2015). Identifying Change Trajectory over the Sumatra’s Forestlands Using Moderate Image Resolution Imagery. Procedia Environ. Sci..

[28] Leinenkugel P., Kuenzer C., Oppelt N., Dech S. (2013). Characterisation of land surface phenology and land cover based on moderate resolution satellite data in cloud prone areas—A novel product for the Mekong Basin. Remote Sens. Environ..

[29] Saadat H., Adamowski J., Tayefi V., Namdar M., Sharifi F., Ale-Ebrahim S. (2014). A new approach for regional scale interrill and rill erosion intensity mapping using brightness index assessments from medium resolution satellite images. CATENA.

[30] Zhang Z., Wang X., Zhao X., Liu B., Yi L., Zuo L., Wen Q., Liu F., Xu J., Hu S. (2014). A 2010 update of National Land Use/Cover Database of China at 1:100000 scale using medium spatial resolution satellite images. Remote Sens. Environ..

[31] Bridhikitti A., Overcamp T.J. (2012). Estimation of Southeast Asian rice paddy areas with different ecosystems from moderate-resolution satellite imagery. Agric. Ecosyst. Environ..

[32] Myint S.W., Gober P., Brazel A., Grossman-Clarke S., Weng Q. (2011). Per-pixel *vs.* object-based classification of urban land cover extraction using high spatial resolution imagery. Remote Sens. Environ..

[33] Soares Machado C.A., Knopik Beltrame A.M., Shinohara E.J., Giannotti M.A., Durieux L., Nóbrega T.M.Q., Quintanilha J.A. (2014). Identifying concentrated areas of trip generators from high spatial resolution satellite images using object-based classification techniques. Appl. Geogr..

[34] Mora B., Wulder M.A., White J.C. (2010). Segment-constrained regression tree estimation of forest stand height from very high spatial resolution panchromatic imagery over a boreal environment. Remote Sens. Environ..

[35] Wania A., Kemper T., Tiede D., Zeil P. (2014). Mapping recent built-up area changes in the city of Harare with high resolution satellite imagery. Appl. Geogr..

[36] Ardila J.P., Bijker W., Tolpekin V.A., Stein A. (2012). Context-sensitive extraction of tree crown objects in urban areas using VHR satellite images. Int. J. Appl. Earth Obs. Geoinf..

[37] Diaz-Varela R.A., Zarco-Tejada P.J., Angileri V., Loudjani P. (2014). Automatic identification of agricultural terraces through object-oriented analysis of very high resolution DSMs and multispectral imagery obtained from an unmanned aerial vehicle. J. Environ. Manag..

[38] Bunting P., Lucas R.M., Jones K., Bean A.R. (2010). Characterisation and mapping of forest communities by clustering individual tree crowns. Remote Sens. Environ..

[39] Pu R., Landry S. (2012). A comparative analysis of high spatial resolution IKONOS and WorldView-2 imagery for mapping urban tree species. Remote Sens. Environ..

[40] Fan Y., Koukal T., Weisberg P.J. (2014). A sun–crown–sensor model and adapted C-correction logic for topographic correction of high resolution forest imagery. ISPRS J. Photogramm. Remote Sens..

[41] Zarco-Tejada P.J., Diaz-Varela R., Angileri V., Loudjani P. (2014). Tree height quantification using very high resolution imagery acquired from an unmanned aerial vehicle (UAV) and automatic 3D photo-reconstruction methods. Eur. J. Agron..

[42] Gomez C., Mangeas M., Petit M., Corbane C., Hamon P., Hamon S., De Kochko A., Le Pierres D., Poncet V., Despinoy M. (2010). Use of high-resolution satellite imagery in an integrated model to predict the distribution of shade coffee tree hybrid zones. Remote Sens. Environ..

[43] Zhou J., Proisy C., Descombes X., le Maire G., Nouvellon Y., Stape J.-L., Viennois G., Zerubia J., Couteron P. (2013). Mapping local density of young Eucalyptus plantations by individual tree detection in high spatial resolution satellite images. For. Ecol. Manag..

[44] Garrity S.R., Allen C.D., Brumby S.P., Gangodagamage C., McDowell N.G., Cai D.M. (2013). Quantifying tree mortality in a mixed species woodland using multitemporal high spatial resolution satellite imagery. Remote Sens. Environ..

[45] Gärtner P., Förster M., Kurban A., Kleinschmit B. (2014). Object based change detection of Central Asian Tugai vegetation with very high spatial resolution satellite imagery. Int. J. Appl. Earth Obs. Geoinf..

[46] Dons K., Smith-Hall C., Meilby H., Fensholt R. (2015). Operationalizing measurement of forest degradation: Identification and quantification of charcoal production in tropical dry forests using very high resolution satellite imagery. Int. J. Appl. Earth Obs. Geoinf..

[47] Cho M.A., Malahlela O., Ramoelo A. (2015). Assessing the utility WorldView-2 imagery for tree species mapping in South African subtropical humid forest and the conservation implications: Dukuduku forest patch as case study. Int. J. Appl. Earth Obs. Geoinf..

[48] Kayitakire F., Hamel C., Defourny P. (2006). Retrieving forest structure variables based on image texture analysis and IKONOS-2 imagery. Remote Sens. Environ..

[49] Radoux J., Defourny P. (2007). A quantitative assessment of boundaries in automated forest stand delineation using very high resolution imagery. Remote Sens. Environ..

[50] Silva T.S.F., Costa M.P.F., Melack J.M. (2010). Spatial and temporal variability of macrophyte cover and productivity in the eastern Amazon floodplain: A remote sensing approach. Remote Sens. Environ..

[51] Lamonaca A., Corona P., Barbati A. (2008). Exploring forest structural complexity by multi-scale segmentation of VHR imagery. Remote Sens. Environ..

[52] Weiers S., Bock M., Wissen M., Rossner G. (2004). Mapping and indicator approaches for the assessment of habitats at different scales using remote sensing and GIS methods. Landsc. Urban Plan..

[53] Sawaya K.E., Olmanson L.G., Heinert N.J., Brezonik P.L., Bauer M.E. (2003). Extending satellite remote sensing to local scales: Land and water resource monitoring using high-resolution imagery. Remote Sens. Environ..

[54] Delenne C., Durrieu S., Rabatel G., Deshayes M. (2010). From pixel to vine parcel: A complete methodology for vineyard delineation and characterization using remote-sensing data. Comput. Electron. Agric..

[55] Antonarakis A.S., Richards K.S., Brasington J. (2008). Object-based land cover classification using airborne LiDAR. Remote Sens. Environ..

[56] Laliberte A.S., Rango A., Herrick J.E., Fredrickson E.L., Burkett L. (2007). An object-based image analysis approach for determining fractional cover of senescent and green vegetation with digital plot photography. J. Arid Environ..

[57] Jacquin A., Misakova L., Gay M. (2008). A hybrid object-based classification approach for mapping urban sprawl in periurban environment. Landsc. Urban Plan..

[58] Conchedda G., Durieux L., Mayaux P. (2008). An object-based method for mapping and change analysis in mangrove ecosystems. ISPRS J. Photogramm. Remote Sens..

[59] Bock M., Xofis P., Mitchley J., Rossner G., Wissen M. (2005). Object-oriented methods for habitat mapping at multiple scales – Case studies from Northern Germany and Wye Downs, UK. J. Nat. Conserv..

[60] Zhang L., Huang X. (2010). Object-oriented subspace analysis for airborne hyperspectral remote sensing imagery. Neurocomputing.

[61] Ouma Y.O., Josaphat S.S., Tateishi R. (2008). Multiscale remote sensing data segmentation and post-segmentation change detection based on logical modeling: Theoretical exposition and experimental results for forestland cover change analysis. Comput. Geosci..

[62] Castillejo-González I.L., López-Granados F., García-Ferrer A., Peña-Barragán J.M., Jurado-Expósito M., de la Orden M.S., González-Audicana M. (2009). Object- and pixel-based analysis for mapping crops and their agro-environmental associated measures using QuickBird imagery. Comput. Electron. Agric..

[63] Ke Y., Quackenbush L.J. Forest species classification and tree crown delineation using Quickbird imagery. Proceedings of the ASPRS 2007 Annual Conference.

[64] Kim S.-R., Lee W.-K., Kwak D.-A., Biging G.S., Gong P., Lee J.-H., Cho H.-K. (2011). Forest Cover Classification by Optimal Segmentation of High Resolution Satellite Imagery. Sensors.

[65] Ghosh A., Joshi P.K. (2014). A comparison of selected classification algorithms for mappingbamboo patches in lower Gangetic plains using very high resolution WorldView 2 imagery. Int. J. Appl. Earth Obs. Geoinf..

[66] Updike T., Comp C. (2010). Radiometric use of WorldView-2 imagery. Tech. Note.

[67] Padwick C., Deskevich M., Pacifici F., Smallwood S. Worldview-2 pan-sharpening. Proceedings of the ASPRS 2010 Annual Conference.

[68] Fienberg S. (1985). The Analysis of Cross-Classified Categorical Data.

[69] Agresti A. (2003). Building and Applying Logistic Regression Models. Categorical Data Analysis.

[70] Menard S.W. (2002). Applied Logistic Regression Analysis. Applied Logistic Regression Analysis.

[71] Ohlmacher G.C., Davis J.C. (2003). Using multiple logistic regression and GIS technology to predict landslide hazard in northeast Kansas, USA. Eng. Geol..

[72] Blaschke T. (2010). Object based image analysis for remote sensing. ISPRS J. Photogramm. Remote Sens..

[73] Breiman L. (2001). Random Forests. Mach. Learn..

[74] Cutler D.R., Edwards T.C., Beard K.H., Cutler A., Hess K.T., Gibson J., Lawler J.J. (2007). Random forests for classification in ecology. Ecology.

[75] Cohen J. (1960). A coefficient of agreement for nominal scales. Educ. Psychol. Meas..

[76] Congalton R.G., Green K. (1999). Assessing the Accuracy of Remotely Sensed Data: Principles and Practices.

[77] Foody G.M. (2004). Thematic map comparison: Evaluating the statistical significance of differences in classification accuracy. Photogramm. Eng. Remote Sens..

[78] Ratle F., Camps-Valls G., Weston J. (2010). Semisupervised Neural Networks for Efficient Hyperspectral Image Classification. Geosci. IEEE Trans. Remote Sens..

[79] Clark M.L., Roberts D.A. (2012). Species-Level Differences in Hyperspectral Metrics among Tropical Rainforest Trees as Determined by a Tree-Based Classifier. Remote Sens..

[80] Immitzer M., Atzberger C., Koukal T. (2012). Tree species classification with Random forest using very high spatial resolution 8-band worldView-2 satellite data. Remote Sens..

[81] Schuster C., Förster M., Kleinschmit B. (2012). Testing the red edge channel for improving land-use classifications based on high-resolution multi-spectral satellite data. Int. J. Remote Sens..

[82] Gitelson A.A., Viña A., Ciganda V., Rundquist D.C., Arkebauer T.J. (2005). Remote estimation of canopy chlorophyll content in crops. Geophys. Res. Lett..

[83] Pathak V., Dikshit O. (2010). A new approach for finding an appropriate combination of texture parameters for classification. Geocarto Int..

[84] Zhong Y., Zhang L. (2012). An Adaptive Artificial Immune Network for Supervised Classification of Multi-/Hyperspectral Remote Sensing Imagery. IEEE Trans. Geosci. Remote Sens..

[85] Yang C., Odvody G.N., Fernandez C.J., Landivar J.A., Minzenmayer R.R., Nichols R.L. (2014). Evaluating unsupervised and supervised image classification methods for mapping cotton root rot. Precis. Agric..

